# Minutes of the 13th Annual Meeting of the Indiana State Dental Association

**Published:** 1871-09

**Authors:** 


### Proceedings of Societies.

### MINUTES OF THE 13th ANNUAL MEETING OF THE INDIANA STATE DENTAL ASSOCIATION.

The association met at the rooms of the Horticultural Society, in Fort Wayne, June 27th, 1871, at 10 o'clock A. M.

President Wm. C. Stanley in the chair.

Members present—J. W. Ellis, J. K. Jameson, P. G. C. Hunt, W. C. Stanley, A. O. Rawls, Isaac Knapp, J. W. Hollingsworth, E. Snider, Jno. F. Johnston, G. W. Loag, Merritt Wells, J. D. Brown, A. M. Moore, Seneca B. Brown, W. H. Gilbert, E. V. Burt, Jno. Crume, S. J. Kirk, W. E. Driscoll, C. C. Burns.

Minutes of last year's meeting were read and approved.

The chair appointed the following Board of Censors—J. K. Jameson, J. D. Brown and E. Snider.

The following persons, having passed a satisfactory examination before the board, were by separate ballot, elected members of the association—Wm. L. Andrews, John Glenn, Burt P. McDonald, W. A. Martin, Charles Manor, Edward H. Creditor, J. R. Clayton.

The Treasurer being absent, J. W. Ellis was appointed Treasurer *pro tem.*

On motion, adjourned to meet at 2 o'clock P. M.

### AFTERNOON SESSION—FIRST DAY.

President Stanley called the meeting to order at 2½ o'clock.

Minutes of morning session read and approved.

On motion of Dr. Knapp, Prof. W. H. Atkinson, of New York; Dr. C. Palmer, of Warren, O.; Dr. Barkley, of

Pittsburg, Pa.; Dr. Barkley, of Ohio, and Dr. Cravens, of Kansas City, Mo., were elected honorary members.

The 1st subject, "What are the Conditions of a tooth which will justify its Extraction," being in order, Dr. Jno. F. Johnston having prepared a paper on the subject, read the same, which received a vote of thanks from the association, and a copy requested for publication. Profs. Atkinson, Moore and Taft, and Dr. Knapp, occupied the remainder of the session in the discussion of Dr. Johnston's paper.

On motion, adjourned to meet at a quarter before 8 o'clock P. M.

### EVENING SESSION—FIRST DAY.

President Stanley resumed the chair at 8 o'clock.

Dr. Snider made some remarks upon the subject in order, when on motion, the discussion was closed, and the second subject, viz: "Treatment of Exposed Nerves" taken up. Dr. Atkinson made a strong appeal for saving all nerve pulps alive, whatever their condition of exposure. Drs. Rawls, Taft and Knapp, occupied the remainder of the session in the discussion of the subject, all condemning the destruction of the nerve pulps in teeth.

The President appointed Profs. Moore, Taft and Atkinson, to operate at the clinic on the following morning. Adjourned.

### MORNING CLINIC—SECOND DAY.

The Professors appointed to operate, were promptly on hand at 8 o'clock, each having their patient in the chair. Prof. Atkinson selected a large posterior proximal cavity, complicated with the crown in an inferior molar; nerve exposed, and gave the association the advantage of witnessing his original and superior manner of filling the same.

Prof. A. M. Moore selected a most difficult case—that of restoring an inferior bicuspid, the posterior half of which was entirely gone. This he accomplished in his usual masterly way, after close application for nearly three hours, much to the admiration and enlightenment of the Society.

Prof. Taft filled a complicated crown cavity. Much benefit is *always* derived from his clinical operations; and the interest manifested at his chair showed this to be no exception.

Most prominent among the new appliances at the clinic, was Dr. Carpenter's Pneumatic Burr Engine. And the numerous orders given for the same, was tangible evidence of the enthusiastic reception the above piece of wonderful mechanism met with, at the hands of the assembled dentists of Indiana.

AFTERNOON SESSION—SECOND DAY.

The President called the association to order at 3½ o'clock.

Secretary read the minutes of the previous session.

The subject of the "Treatment of Exposed Nerves" was resumed. Dr. Taft opened the discussion, pleading earnestly for more faith in treating these cases. Drs. Snider, Rawls, and Knapp followed. Prof. Atkinson said that he congratulated himself, that he heard fifty dentists in the Hoosier State, discussing those deep principles of physiology and pathology, and thus laying solid foundations for future achievements. He plead for the respect of individual judgment in these cases, denying the power of one to dictate to another, it matters not what his position may be; he stated that there were no two cases in this class alike, and each one stood alone *sui generis*, and must be diagnosed and prescribed for by itself.

On motion, the society adjourned, to meet at 8 o'clock, in the room of Circuit Court, for a public discussion.

EVENING SESSION—SECOND DAY.

The association convened at 8 o'clock P. M., pursuant to adjournment, in the rooms of the Circuit Court, to which the public had been invited to listen. The 4th subject in the order of discussion was here taken up, namely, "Can the Decay of Teeth be Prevented."

Prof. Taft opened the debate, taking strong grounds in the affirmative of the question.

He traced the causes of decay to almost every physical irregularity, to careless and vicious habits as regards health, and to neglect of proper care by the young. Every disease, even those apparently the farthest removed from apparent connection with the teeth, has a greater or less effect upon these organs. He made a very earnest plea for increased attention to children's first teeth.

Prof. W. H. Atkinson followed. He took as his text, "Live Godly, Soberly and Righteous Lives," and PREACHED a masterly discourse, setting forth vividly the great duty of redemption of the body, and thus paying due respect to our Creator.

At the conclusion of Dr. A's remarks, the association adjourned, until 8 o'clock the following morning. Dr. Atkinson having been appointed to hold a clinic at that hour.

### MORNING CLINIC—THIRD DAY.

Prof. Atkinson conducted the clinic agreeable to appointment. The operation was that of removing accumulations of lime from the teeth, (wrongfully called tartar). After removing as thoroughly as possible with instruments, aromatic elixir of vitriol was applied to dissolve remaining particles, care being taken to protect the teeth. During the operation Dr. Atkinson explained to the association, his treatment to restore the wasted tissues, both bony and soft, and many other valuable hints in regard to cleansing teeth.

### MORNING SESSION—THIRD DAY.

The President called the association to order at 11 o'clock. After the reading of the minutes of last session, the regular order of business was, on motion, set aside, and "Miscellaneous" taken up.

On motion of Dr. Johnston, a Committee on Appliances was appointed by the chair, consisting of Drs. Moore, Knapp and Hunt.

Dr. Hunt moved the Financial Committee authorize the Secretary to draw upon the Treasurer, for funds to defray all necessary expenses attending this association. Carried.

On motion of Dr. Knapp, the association proceeded to the election of officers for the ensuing year.

The election resulted as follows:

*President*—Dr. P. G. C. Hunt, of Indianapolis.
1*st. Vice President*—A. O. Rawls, of Connersville.
2*d. Vice President*—Wm. L. Andrews, of Kendallville.
*Secretary*—Burt P. McDonald, of Goshen.
*Treasurer*—Isaac Knapp, of Fort Wayne.

Indianapolis was selected as the next place of meeting.

An Auditing Committee, consisting of Drs. Johnston, Burt and S. B. Brown, having been appointed, made their report through their chairman, Dr. Johnston, on the finance of the society, which was accepted and the committee discharged.

The thanks of the association were then tendered to the dentists and citizens of Fort Wayne, for their kindness and hospitable attention to the association while in session.

On motion, Dr. E. R. E. Carpenter, of Chicago, and Dr. John L. Scott, of Defiance, Ohio, were elected to honorary membership.

The association then adjourned until 2 o'clock P. M.

### AFTERNOON SESSION—THIRD DAY.

The association convened at 2 o'clock. President Stanley in the chair.

The Committee on Appliances, through their chairman, Dr. Hunt, made their report. The pneumatic burr, drill, file carrier, polisher, universal joint, and automatic mallet, received their entire approval.

On motion, the report was accepted, and committee discharged. Prof. Atkinson, wished to add his testimony in the strongest terms in favor of the Carpenter Pneumatic Engine, and took the position that no dentist doing even a moderate business, could afford to do without it.

President Stanley, retiring from the chair, delivered his address, and as his last official act appointed Drs. Moore and Jameson, to conduct the President elect, Dr. Hunt, to the chair. He accepted the honor in a brief address.

President Hunt then announced that the third topic for discussion, "Filling Teeth," (proximal cavities of bicuspids) was in order.

The discussion upon this subject was participated in by Drs. Atkinson, Taft, Palmer, Knapp and Rawls.

Dr. Palmer, of Warren, Ohio, concluded the discussion on the subject, giving the association the benefit of his wonderful skill, and system in filling teeth. He also exhibited fillers of his own make, which were models of beauty and perfection.

On motion, the regular order of discussion was dispensed with, and the "Miscellaneous Business" taken up.

The Secretary read communications from Drs. Geo. W. Keeley, of Oxford, Ohio; G. A. Mills, of Brooklyn, New York, and A. T. Keightly, of Green Castle, Ind., who expressed their regrets at not being able to be present, and extended their best wishes for a pleasant gathering of the association. Also a paper from Dr. W. F. Morrill, of New Albany, now traveling in Brazil, on the subject of "Dentistry in Brazil."

President Hunt appointed as Executive Committee for the ensuing year, Drs. John F. Johnston, Wm. L. Andrews and E. V. Burt.

Dr. Knapp read a proposition from Dr. A. Wells, of Fort Wayne, offering for a consideration, to give to the dentists of the United States, what knowledge he possesses of the construction, repairing, etc., of "porcelain work."

Dr. Johnston, moved the proposition be given publicity in the DENTAL REGISTER. Carried.

Miscellaneous business was concluded, and the fifth subject, "Ethics" taken up, and an essay from Dr. E. M. Morrison was read, which received the thanks of the society and ordered published. Also the paper from Dr. Morrill and Dr. Stanley's address.

The association then adjourned, to meet in Indianapolis, the last Tuesday in June, 1872.

SENECA B. BROWN, *Secretary.*

*To the Indiana State Dental Association, now in Convention at Fort Wayne:*—The undersigned would respectfully submit the following proposition, to wit:

That if the said convention, or the dentists of the United States, will raise him $1,000, he will instruct any practical dentist designated, in all the particulars, how to make and set and mend the sets of porcelain teeth, the same as he makes and sets and mends them; and also to make all the materials and enamels that he uses in doing the same. He would also request permission to state the opinion that to know how to mend the said porcelian teeth and plates when broken, would be of more value to the world than the amount mentioned.

All of which is respectfully submitted.

A. WELLS.

